# Reducing Pessimism About Social Security’s Future Through Gerontological Education

**DOI:** 10.1093/geroni/igab046.1510

**Published:** 2021-12-17

**Authors:** Catheryn Koss

**Affiliations:** California State University, Sacramento, Sacramento, California, United States

## Abstract

A recent Pew Research Center poll found less than half of non-retired U.S. adults believe they will receive Social Security benefits when they retire. Pessimism about Social Security’s future is even stronger among people under 50, raising concerns about political support for the program. The current study examined to what extent education about how Social Security functions and strategies to address program funding shortfalls could increase optimism about Social Security’s future. In Fall 2020, twenty-two undergraduate students enrolled in an on-line aging and social policy course were asked how likely they would receive Social Security income when they retired. Consistent with national polling data, only 38% believed they would probably or definitely receive Social Security retirement income. Students were also asked to briefly explain why they answered the way they did. Common reasons given for pessimism about receiving Social Security retirement income included having heard Social Security will run out of money and believing only U.S. citizens are eligible for Social Security benefits. Over a two-week period, students completed asynchronous on-line activities covering Social Security retirement eligibility, worker and spousal benefits, current funding, and proposed changes to address funding shortfalls. Upon completion, 67% of students stated they would probably or definitely receive Social Security retirement income when they retired (p = .07). These preliminary results suggest that education about the program can reduce pessimism about Social Security’s future. Additional data will be collected from 45 students enrolled in the same course in Spring 2021.

